# Enterocyte-Based Bioassay via Quantitative Combination of Proinflammatory Sentinels Specific to 8-keto-trichothecenes

**DOI:** 10.3389/fimmu.2020.01530

**Published:** 2020-07-16

**Authors:** Seong-Hwan Park, Yuseok Moon

**Affiliations:** ^1^Laboratory of Mucosal Exposome and Biomodulation, Department of Biomedical Sciences, Pusan National University, Yangsan, South Korea; ^2^Biomedical Research Institute, Pusan National University, Yangsan, South Korea

**Keywords:** 8-keto-trichothecene, ribotoxic stress, inflammation, epithelium, reporter biomonitoring

## Abstract

Type B 8-keto-trichothecenes are muco-active mycotoxins that exist as inevitable contaminants in cereal-based foodstuffs. Gut-associated inflammation is an early frontline response during human and animal exposure to these mycotoxins. Despite various tools for chemical identification, optimized biomonitoring of sentinel response-associated biomarkers is required to assess the specific proinflammatory actions of 8-keto-trichothecenes in the gut epithelial barrier. In the present study, intoxication with 8-keto-trichothecenes in human intestinal epithelial cells was found to trigger early response gene 1 product (EGR-1) that plays crucial roles in proinflammatory chemokine induction. In contrast, epithelial exposure to 8-keto-trichothecenes resulted in downregulated expression of nuclear factor NF-kappa-B p65 protein, a key transcription factor, during general inflammatory responses in the gut. Based on the early molecular patterns of expression, the inflammation-inducing activity of 8-keto-trichothecenes was quantified using intestinal epithelial cells with dual reporters for EGR-1 and p65 proteins. EGR-1-responsive elements were linked to luciferase reporter while p65 promoter was bound to secretory alkaline phosphatase (SEAP) reporter. In response to conventional inflammagens such as endotoxins and cytokines such as TNF-α, both luciferase and SEAP activity were elevated in a dose-dependent manner. However, as expected from the mechanistic evaluation, 8-keto-trichothecene-exposed dual reporters of luciferase and SEAP displayed contrasting expression patterns. Furthermore, 8-keto-trichothecene-elevated EGR-1-responsive luciferase activity was improved by deficiency of PSMA3, an α-type subunit of the 20S proteasome core complex for ubiquitin-dependent EGR-1 degradation. This molecular event-based dual biomonitoring in epithelial cells is a promising supplementary tool for detecting typical molecular inflammatory pathways in response to 8-keto-trichothecenes in the food matrix.

## Introduction

Trichothecenes are a group of over 200 sesquiterpenoid fungal metabolites, some of which are health-threatening mycotoxins leading to eukaryotic ribosome stalling (1–4) or cellular ubiquitin depletion (5, 6). Extensive investigation into toxic trichothecenes have been made due to their widespread contamination in the environment (7, 8) and agricultural commodities such as grains (9–11). Moreover, some of trichothecenes are released from mold-infected field crops into surface soil water and drainage systems (7, 8). Trichothecenes are characterized by a tetracyclic scirpenol ring system and are classified into four groups (types A–D), depending on the presence of the functional moiety (12, 13). Field fungi such as *Fusarium* species mainly produce type A and B trichothecenes. Type A trichothecenes are characterized by a hydroxyl or acyl moiety at the R1, R2, R3, or R5 position of the tetracyclic scirpenol ring. The Type B group toxins are typically named as 8-keto-trichothecenes due to a ketone group at R5. The ribosome-inactivating activity of trichothecenes has been linked to the presence of an intact 9,10 double bond, C-12,13 epoxide, and substitution of both R1 and R2 (13–16).

Epidemiological investigations have demonstrated positive associations between human exposure to trichothecene and gastrointestinal disorders including gastroenteritis and esophageal cancer (17–19). In particular, the 8-keto-trichothecenes such as deoxynivalenol (DON) and nivalenol (NIV) can produce a variety of mycotoxicosis in humans and animals. Acutely intoxicated animals display severe injuries in gastrointestinal tract and highly-proliferating immune-related cells, leading to diarrhea, vomiting, gastrointestinal hemorrhage ad leukocytopenia (10, 20–22). In addition to the direct tissue injuries, DON-exposed mice show cytokine storm syndromes during mucosal and systemic intoxication (23–26). Epithelial responses are the early gastrointestinal event in response to diet-derived contaminants. The gut epithelial linings sense luminal insults and facilitate molecular reprograming during mucosal and systemic exposure to trichothecenes (27–29). The epithelial barrier responds to luminal insults that activate stress-responsive cellular signaling such as mitogen-activated kinases or other pathologic biochemical pathways. The activated stress kinases then alter profiles of immediately responsive genes such as early growth response gene 1 (*EGR-1*), which contribute to maintaining tissue integrity during pathologic states by itself or through its regulated gene products (30–32). Moreover, in response to mucosal microbial antigens, pattern recognition receptors are activated and transmit danger signals via proinflammatory transcription factors mainly including nuclear factor-κB (NF-κB) (33, 34). EGR-1 is also involved in diverse inflammatory cellular events including inflammatory cell migration and cytokine production (35–38).

In spite of a variety of detection methods for trichothecene metabolites, conventional analytical technologies are often insufficient to identify their biological activity in the contaminated matrix. We thus developed an enterocyte-based bioassay system measuring epithelial stress responses to 8-keto-trichothecenes. In the present study, we evaluated EGR-1 as a crucial biomarker in the epithelial exposure to 8-keto-trichothecenes. An optimized EGR-1-reporting system was constructed in human intestinal cells by combining with a representative inflammatory biomarker, NF-κB, in response to the unavoidable trichothecenes. This challenge could provide useful biomaterials to measure the early biological activities of proinflammatory trichothecenes in food, feed, and the environment.

## Materials and Methods

### Cell Culture Conditions and Reagents

The intestinal epithelial cell line, HCT-8 (passage 13), was purchased from ATCC (Rockville, MD, USA). Based on our previous report (39), HCT-8 cells were maintained in RPMI 1640 medium (Welgene, Daegu, South Korea) supplemented with 10% (v/v) heat-inactivated fetal bovine serum (FBS, Welgene), 50 units/ml penicillin and 50 μg/ml streptomycin (Welgene) in a 5% CO_2_ humidified incubator at 37°C. Cell counting was performed by trypan blue dye exclusion (Sigma-Aldrich chemical company, St. Louis, MO, USA) using a hemocytometer. All other chemicals were obtained from Sigma-Aldrich except for TNF-α (Peprotech, Rocky Hill, NJ, USA).

### Mouse Experiments

C57BL/6 mice (6 weeks old with an average weight of 16–18 g) were purchased from Jackson Laboratories (Bar Harbor, ME, USA). Based on our previous report (40), mice were acclimated for 14 days prior to the experiments and maintained at 25°C and 45–55% relative humidity under 12-h light/dark cycles. Mice were housed three per cage and provided sufficient food and water. Animal care and experimental procedures were conducted in accordance with the guidelines of the Institutional Animal Care and Use Committee. Mice were maintained in environmentally protected cages consisting of a transparent polypropylene body and a stainless-steel wire top cover. This animal study was approved by the Pusan National University Institutional Animal Care and Use Committee (PNU-IACUC) (PNU-2013-0291).

### Immunohistochemistry

Based on our previous report (41), formalin-fixed paraffin-embedded tissues were cut, deparaffinized, and rehydrated. The tissue sections were heated in 10 mM sodium acetate (pH 9.0) for 5 min at 121°C for antigen retrieval. To remove endogenous peroxidase, tissues were bathed in a 1% (v/v) H_2_O_2_-PBS solution for 15 min at room temperature in the dark. The samples were then washed with 0.1% TBS-T, and blocked with 3% (w/v) FBS in PBS for 1 h. The blocked samples were incubated overnight at 4°C with the primary antibodies (diluted at 1:200). After washing three times with 0.1% TBS-T, samples were incubated with the horseradish peroxidase-conjugated secondary antibody (diluted at 1:200) for 2 h at room temperature and then washed three times with 0.1% TBS-T. The bound antibodies were identified using freshly prepared substrate buffer (0.05% [w/v] diaminobenzidine (DAB; Sigma-Aldrich) and 0.01% [v/v] H2O2 in PBS) for 5 min. After a final wash in distilled water, the sections were counterstained with hematoxylin (Bioworld, Dublin, Ohio, USA) solution for 1 min and dehydrated. Sections were examined at various magnifications using an Eclipse Ts2R (Nikon Instruments Inc., Melville, NY, USA). The ratio of the target protein-expressing cells was measured using HistoQuest software (TissueGnostics, Vienna, Austria).

### Western Immunoblot Analysis

Protein expression levels were compared by western immunoblot analysis using rabbit polyclonal anti-β-actin (actin) antibody, rabbit polyclonal anti-EGR-1, rabbit polyclonal anti-NF-κB p65 (Santa Cruz Biotechnology, Santa Cruz, CA, USA), and rabbit polyclonal phosphor-NF-κB p65 (Cell Signaling Technology, Beverly, MA, USA). Based on our previous report (42), the cells were washed with ice-cold phosphate buffer, lysed in boiling lysis buffer [1% (w/v) SDS, 1.0 mM sodium *ortho*-vanadate, and 10 mM Tris, pH 7.4], and sonicated for 5 s. Lysates containing proteins were quantified using a BCA protein assay kit (Welgene). Next, 15 μg of proteins were separated by Bio-Rad mini gel electrophoresis, and transferred to a PVDF membrane (Pall Corporation, Port Washington, NY, USA). The blots were then blocked for 1 h with 5% skimmed milk in Tris-buffered saline plus Tween 0.1% (TBST). The samples were probed with each antibody (diluted at 1:1,000) for an additional 2 h at room temperature or overnight at 4°C. After washing three times with TBST, blots were incubated with horseradish-conjugated secondary antibody (diluted at 1:5,000) for 2 h and then washed thrice with TBST. Finally, the proteins were detected using a pico-enhanced peroxidase detection system (ELPIS Biotech., Inc., Taejon, South Korea).

### Reverse Transcription and Real Time PCR

Based on our previous reports (40, 41), RNA was extracted using RiboEx (GeneAll Biotech, Seoul, South Korea) according to the manufacturer's instructions. RNA (3 μg) from each sample was transcribed to cDNA using Prime RT premix (Genetbio, Nonsan, South Korea). During real time PCR, 6-carboxyl fluorescein was used as the fluorescent reporter dye to detect the amplified cDNA. Real time PCR was conducted using an iCycler Thermal Cycler (Bio-Rad) with the following conditions: denaturation at 95°C for 15 min followed by cycles of denaturation at 95°C for 20 s, annealing at 59°C for 30 s, and elongation at 72°C for 30 s. Each sample was tested in triplicate to ensure statistical significance. Quantification of relative gene expression was performed using the comparative *Ct* method. The *Ct-*value is defined as the point at which a statistically significant increase in fluorescence occurs. The number of PCR cycles (*Ct*) required for the 6-carboxylfluorescein intensities to exceed the threshold of just above the background level was calculated for the test and reference reactions. In all experiments, GAPDH was used as the endogenous control. The 5′ forward and 3′ reverse PCR primers for amplifying each gene were as follows: human p65, 5′-GGC GAG AGG AGC ACA GAT AC-3′ and 5′-ATC TTG AGC TCG GCA GTG TT-3′; human PSMA3, 5′-GCT CAA TCG GCA CTG GGT AT-3′ and 5′-GGC CACA TCT GTC TGC AAG AT-3′; and human GAPDH, 5′-TCA ACG GAT TTG GTC GTA TT-3′ and 5′-CTG TGG TCA TGA GTC CTT CC-3′.

### Construction of Plasmids

Empty vector or shRNA of PSMA3 (shPSMA3) were constructed using the pMKO.1 GFP vector purchased from Addgene (a gift from William Hahn). The inserted PSMA3 shRNA targeted the sequence: 5′-TAA AGC TTT TGA ACT AGA A-3′. The luciferase reporter construct, pEBS1^4^-LUC plasmid, which contained four EGR-1 binding sites, was provided by Dr. Seung-Joon Baek (University of Tennessee). Human p65 promoter (−999/+92) was generated by RT-PCR using human genomic DNA from HCT-8 cells with the following primers: 5′-AAG ACG AAT TCA GAG AAA ATA AAA A-3′ and 5′-TGC ACT ACA GAC GAG CCA TT-3′. The resulting 1,091 bp construct was cloned by Kpn1/Xho1 excision, and inserted in the sense orientation into the reporter plasmid, pGL4.14-SEAP-hygro, in which the luciferase gene cassette was replaced with the SEAP gene cassette through HindIII and HpaI excision followed by blunt end formation using DNA polymerase I (New England Biolabs, Ipswich, MA, USA). The flag-tagged SR-IkB expression vector has been described previously (43).

### Luciferase Assay

Based on our previous report (40), cells were washed with cold PBS, lysed with passive lysis buffer (Promega), and then centrifuged at 13,800 × g for 10 min. The supernatant was collected and stored at −80°C until measurement. Luciferase activity was measured with a model TD-20/20 dual mode luminometer (Turner Designs, Sunnyvale, CA) after briefly mixing the supernatant (10 μl) with 40 μl of firefly luciferase assay substrate solution, followed by stopping with 50 μl of *Renilla* luciferase stop solution (Promega). The firefly luciferase activity was divided by the *Renilla* luciferase activity for normalization.

### Secreted Alkaline Phosphatase (SEAP) Assay

Transformed HCT-8 cells were seeded in a 24-well-plate at a density 5 × 10^4^ cells/well and were then treated with various concentrations of mycotoxins and other chemicals for 24 or 72 h at 37°C. Based on our previous report (13), the collected culture medium (400 μl) was heated at 65°C for 5 min and then 100 μl of the heated supernatant was mixed with 100 μl of 2× SEAP assay buffer (2M diethanolamine, 1 mM MgCl_2_, 20 mM L-homoarginine). The mixture was incubated at 37°C for 10 min and the reaction was terminated by adding 20 μl of 120 mM p-nitrophenylphosphate dissolved in 1× SEAP assay buffer. The final mixture was further incubated at 37°C in the dark. The absorbance of the reaction mixture was measured at 405 nm using an enzyme-linked immunosorbent assay (ELISA) reader (Molecular Devices, Sunnyvale, CA, USA).

### Statistics and Reproducibility

Statistical analyses were performed using GraphPad Prism v. 8.01 (La Jolla, CA, USA). For comparative analysis of two groups of data, Student's *t-*test was performed. For comparative analysis of multiple groups, data were subjected to analysis of variance (ANOVA) with *Newman-Keuls* method as a *post-hoc* ANOVA assessment. All *in vitro* evaluations are representative of two or three independent experiments. Details of the number of biological replicates and the assays are given in each figure legend.

## Results

### 8-keto-trichothecenes Induce EGR-1 and Chemokine Expression in Intestinal Epithelial Cells

**Figure 1:**
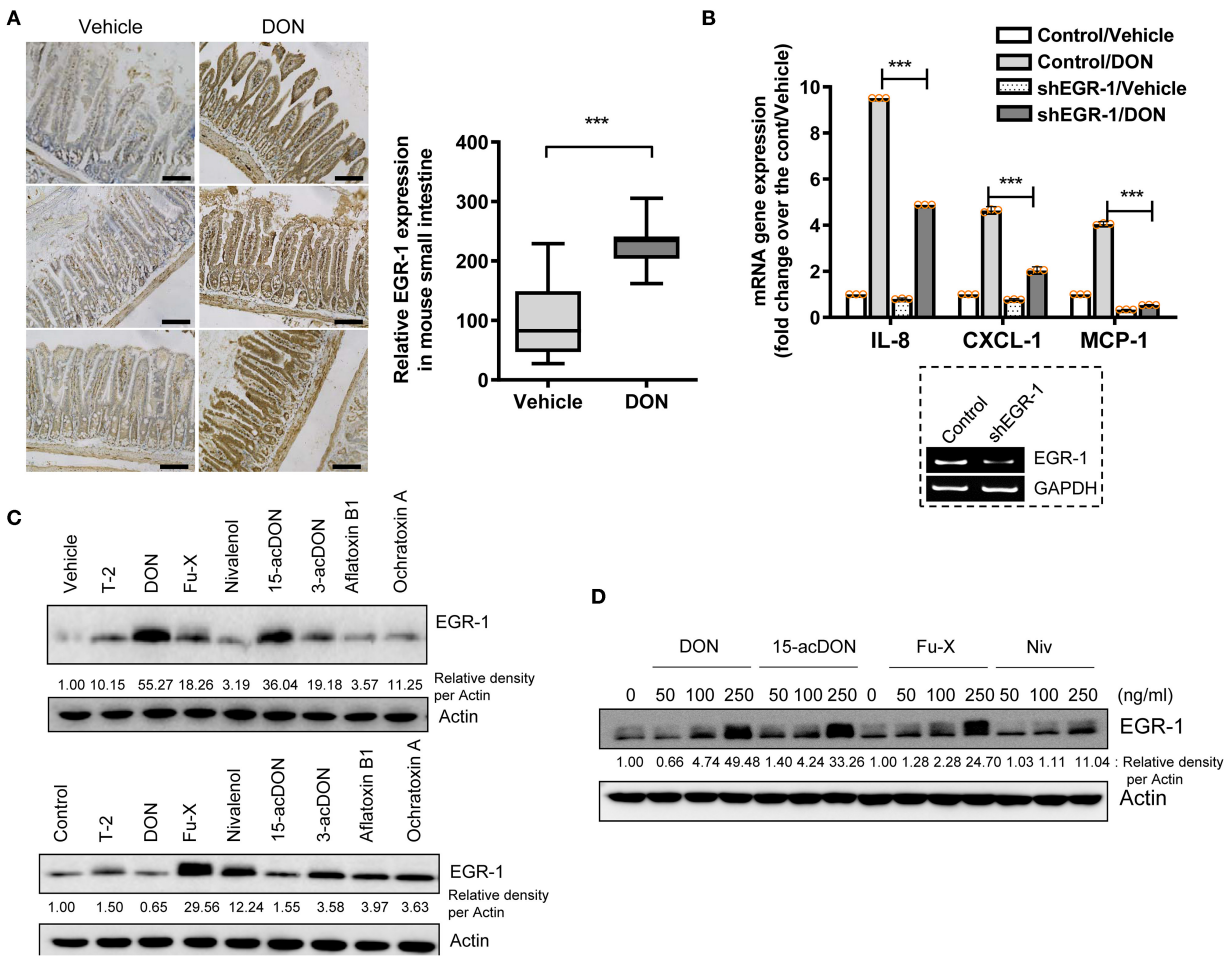
Exposure to 8-keto-trichothecenes induces gut EGR-1 expression. **(A)** Small intestinal tissues from 6-week-old male mice exposed to vehicle or 25 mg/kg DON for 12 h were subjected to IHC analysis for EGR-1 (*n* = 4, each group; original magnification, ×200, black scale bar, 0.2 μm). Right panel shows the quantitative analysis of tissue EGR-1 levels in vehicle or DON-treated mice. Results are shown as box-and-whisker plots (min–max) and asterisks (***) indicate significant difference from the control (*p* < 0.001). **(B)** Control- or shEGR-1-transfected HCT-8 cells were treated with vehicle or 500 ng/ml DON for 1 h. mRNA expression was measured using RT real-time PCR. Results are shown as mean values ± SD and asterisks (***) indicate a significant difference (*p* < 0.001, *n* = 3). The figure in the box represents the relative expression of EGR-1 with vehicle or shEGR-1. **(C)** HCT-8 cells were treated with vehicle or each mycotoxin at 20% cell growth-inhibitory doses (ID_20_) (3 ng/ml T-2 toxin, 500 ng/ml DON, 400 ng/ml Fu-X, 300 ng/ml NIV, 300 ng/ml 15-acDON, 2,500 ng/ml 3-acetyl DON, 1 μM aflatoxin B1, or 1 μM Ochratoxin A) for 1.5 h (upper panel) or 5 h (lower panel). **(D)** HCT-8 cells were exposed to each dose of DON and 15-acDON for 1.5 h and to each dose of Fu-X and NIV for 5 h. Total cell lysates were subjected to western blot analysis.

The proinflammatory actions of 8-keto-trichothecenes initiate the deleterious progress of acute and chronic diseases in humans and domestic animals. In response to toxic insult, the stress-responsive transcription factor EGR-1 was assessed as an early trigger of inflammatory gene expression. Gastrointestinal exposure to DON, the most prevalent trichothecene in the food chain, caused notable induction of the EGR-1 protein in the gut epithelial layer compared to that in the lamina propria (Figure 1A). Next, we assessed the proinflammatory chemokine expression in trichothecene-exposed HCT-8 cells, which are widely used human intestinal epithelial cells (IEC) to model inflammatory and infectious diseases (44, 45). In particular, the ileocecum of the small intestine, which is the source of the HCT-8 cell line, is one of the regions that is most susceptible to ribosomal inactivation-associated xenobiotic stress (46, 47). EGR-1 is a crucial transcription factor of DON-induced proinflammatory cytokines, as proven by the expression of interleukin-8 (IL-8), chemokine (C-X-C motif) ligand 1 (CXCL1), and monocyte chemoattractant protein-1 (MCP-1) in human IECs (Figure 1B). In addition to DON, other trichothecenes and mycotoxins were also compared for their effects on EGR-1 expression in HCT-8 cells.

Other mycotoxins were assessed for their effects on EGR-1 expression in human IECs at 20% cell growth-inhibitory doses (ID_20_) and optimal induction times (1.5 or 5 h treatment). In particular, 8-keto-trichothecenes such as DON, fusarenon-X or 4-acetyl nivalenol (Fu-X), 15-acetyl DON (15-acDON), and NIV were among the strongest agents to induce EGR-1 expression (Figure 1C). Furthermore, we evaluated the dose responses of EGR-1 induction by these 8-keto-trichothecenes. Treatment with 8-keto-trichothecenes induced EGR-1 expression in human IECs in a dose-dependent manner (Figure 1D). In subsequent experiments, EGR-1 was assumed as a potent early biomarker of proinflammatory insult in response to 8-keto-trichothecenes.

### PSMA3 Deficiency Enhanced 8-keto-trichothecene-induced EGR-1 Levels in IECs

**Figure 2:**
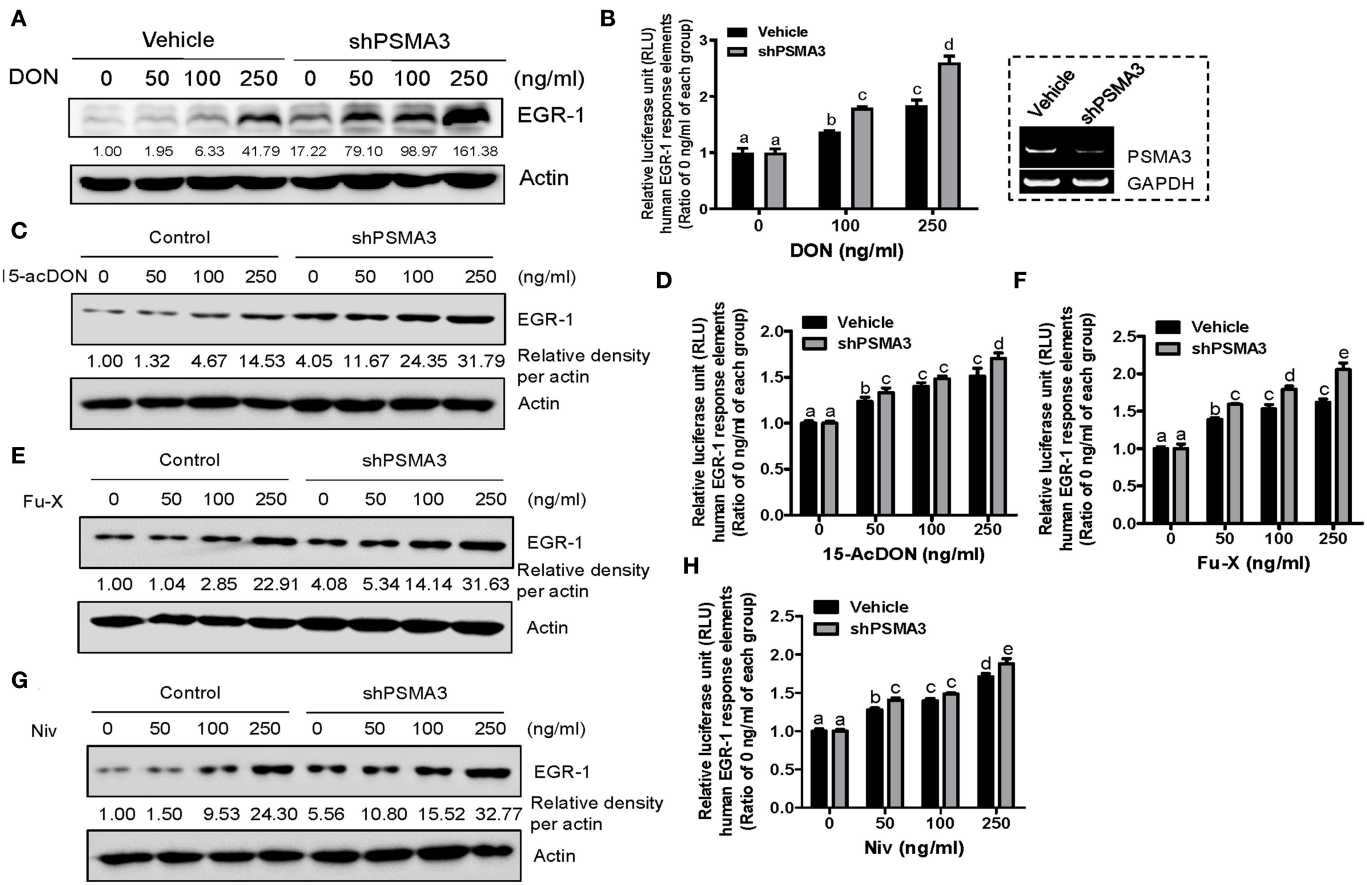
PSMA3 counteracts EGR-1 expression in IECs exposed to 8-keto-trichothecenes. **(A,C,E,G)** Control or PSMA3-deficient HCT-8 cells were treated with each dose of 8-keto-trichothecene for 1.5 h **(A,C)** or 5 h **(E,G)**. Total cell lysates were subjected to western blot analysis. **(B,D,F,H)** Control or PSMA3-deficient HCT-8 cells were transfected with pEBS1^4^-LUC plasmid and treated with each dose of 8-keto-trichothecene for 24 h. The figure in the box represents the efficiency of shPSMA3 expression. Results are shown as mean values ± SD and different letter over each bar represents a significant difference between the groups (*p* < 0.05, *n* = 3).

To maintain persistent levels of induced EGR-1 as a crucial readout of 8-keto-trichothecene toxicity, its protein stability needs to be improved. Biochemically, proteasome subunit alpha type-3 (PSMA3)/proteasome component C8 (PRC8) is the α-type subunit of the 20S proteasome core complex that interacts with EGR-1, leading to its ubiquitin-dependent proteasome degradation (48). Therefore, we assessed whether PSMA3 deficiency improves 8-keto-trichothecene-induced EGR-1 expression in IECs. PSMA3-suppressed IECs displayed enhanced levels of EGR-1 expression in response to DON exposure (Figure 2A). To evaluate EGR-1-mediated transcriptional regulation, we applied a luciferase reporter construct, pEBS1^4^-LUC plasmid, which contained EGR-1 binding sites. Furthermore, DON-induced EGR-1-mediated transcriptional activity was enhanced in stable PSMA3-suppressed IECs (Figure 2B). Other 8-keto-trichothecenes including 15-acDON, Fu-X, and NIV also increased EGR-1 protein levels (Figures 2C,E,G) and transcription activity (Figures 2D,F,H) in a dose-dependent manner, which was enhanced in PSMA3-deficient IECs compared to the levels in control vector-transfected IECs. In conclusion, regulation of PSMA3 action improved the EGR-1-mediated responses to 8-keto-trichothecenes in human IECs.

### NF-κB Subunit Expression Is Suppressed by 8-keto-trichothecenes in IECs

**Figure 3:**
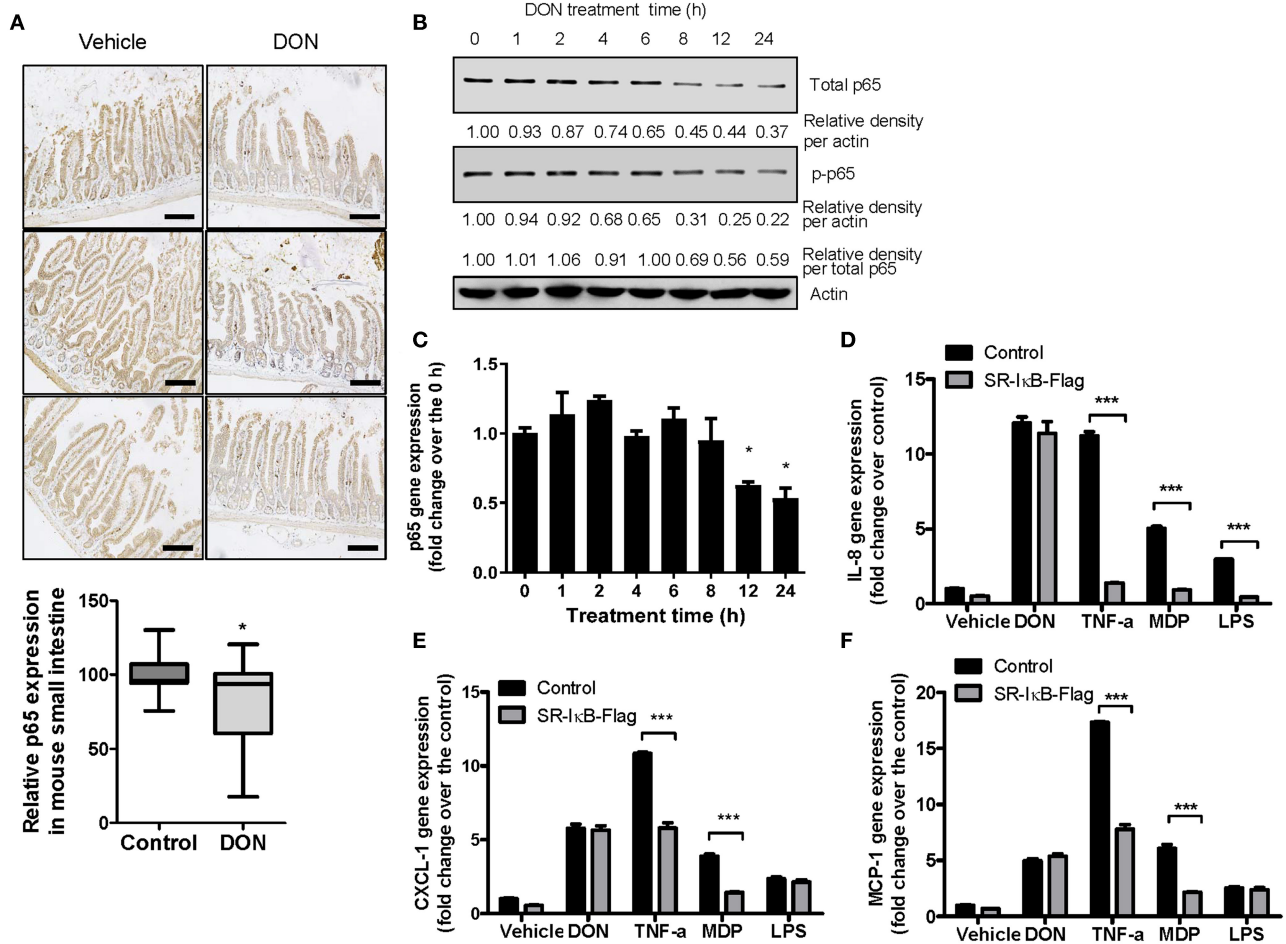
Roles of p65 in the expressions of p65 and chemokines in response to DON or inflammagens in IECs. **(A)** Small intestinal tissues from 6-week-old male mice exposed to vehicle or 25 mg/kg DON for 12 h were subjected to IHC analysis for p65 (*n* = 4, each group) (original magnification, × 200, black scale bar, 0.2 μm). Lower panel shows the quantitative analysis of tissue p65 levels in vehicle or DON-treated mice. Results are shown as box-and-whisker plots (min–max) and asterisk (*) indicates a significant difference from the control (*p* < 0.05, *n* = 3). **(B,C)** HCT-8 cells were treated with 500 ng/ml DON for the indicated time. Cell lysates were subjected to western blot analysis **(B)** and mRNA expression was measured using RT real-time PCR **(C)**. Asterisk (*) indicates a significant difference from the 0 h group (*p* < 0.05). **(D–F)** HCT-8 cells transfected with control or SR-IκB-Flag were treated with vehicle, 500 ng/ml DON, 10 ng/ml TNF-α, 10 μg/ml MDP, or 2 μg/ml LPS for 1 h. IL-8 **(D)**, CXCL-1 **(E)**, and MCP-1 **(F)** mRNA was measured using RT real-time PCR. Results are shown as mean values ± SD and asterisks (***) indicate a significant difference from each control group (*p* < 0.001, *n* = 3).

Although DON is known to trigger inflammatory responses, the levels of p65 as a subunit of NF-κB were significantly reduced by DON treatment in gut epithelia (Figure 3A), consistent with our previous report on p65 suppression by DON at the cellular level (49). DON-treated IECs showed reduced levels of total and phosphorylated p65 (Figure 3B) as well as p65 mRNA (Figure 3C). Since NF-κB is a representative proinflammatory transcription factor in responses to cytokines or microbial components, inhibition of NF-κB signaling significantly attenuated the chemokine induction in response to tumor necrosis factor-α (TNF-α), or other endotoxins such as muramyl dipeptide (MDP) and lipopolysaccharide (LPS) (Figures 3D–F). NF-κB signaling was suppressed using an NF-κB inhibitory construct, super-repressor (SR)-IκB, which expresses a mutated form of IκB, resulting in inhibition of IκB phosphorylation and degradation. In contrast, SR-IκB did not affect the levels of DON-induced proinflammatory chemokines in human IECs (Figures 3D–F). Taken together, DON suppresses p65 mRNA and protein levels even though the toxin induces proinflammatory chemokines in IECs.

### PSMA3 Deficiency Does Not Affect p65 Expression in Response to 8-keto-trichothecenes

**Figure 4:**
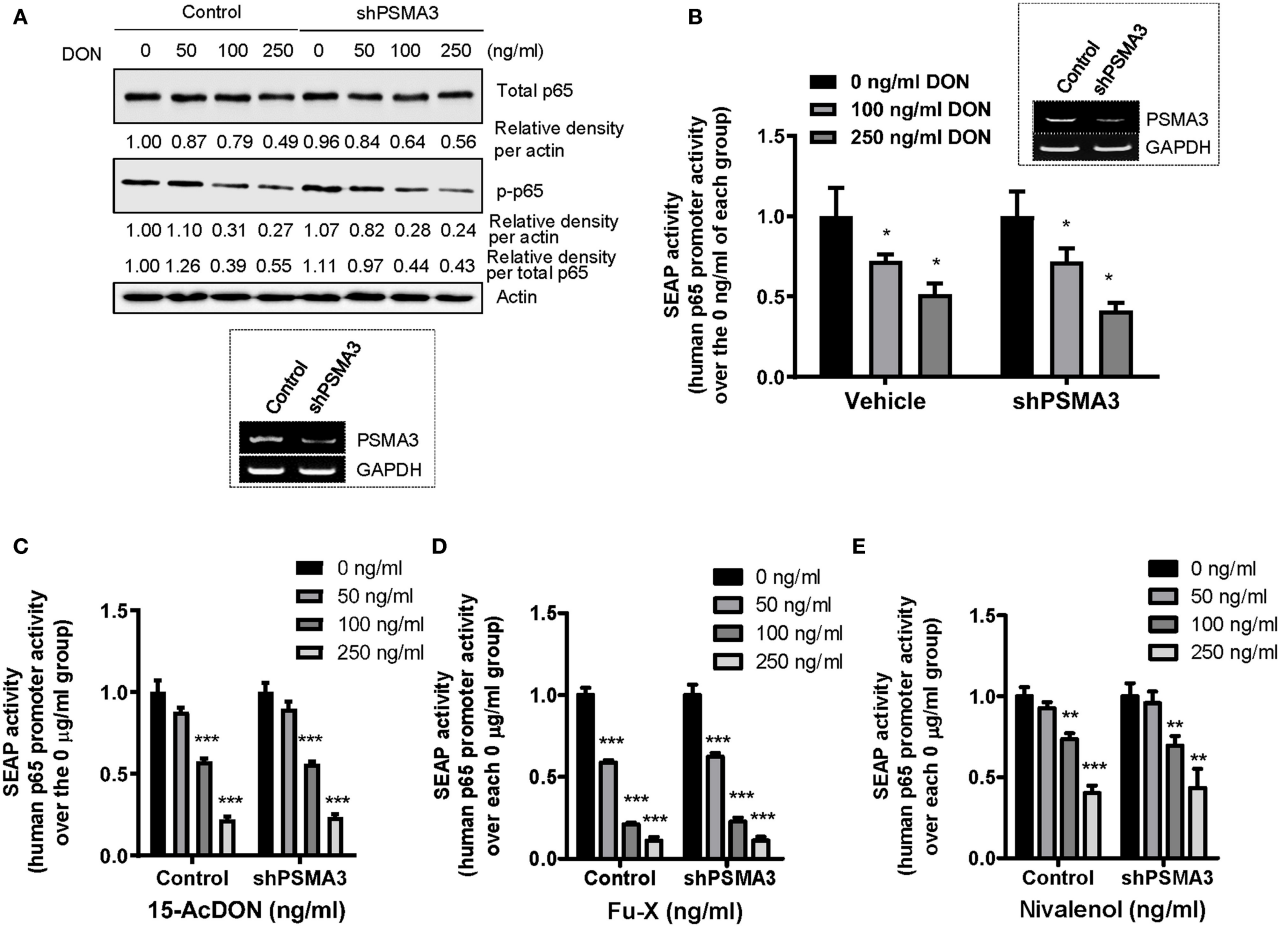
Effects of PSMA3 deficiency on p65 expression in response to 8-keto-trichothecenes. **(A)** Control- or PSMA3-deficient HCT-8 cells were treated with each dose of DON for 9 h. Cell lysates were subjected to western blot analysis. The figure in the box represents the efficiency of PSMA3 shRNA expression. **(B–E)** Control- or PSMA3-deficient HCT-8 cells were transfected with human p65 promoter-containing SEAP reporter plasmid and were treated with each dose of 8-keto-trichothecenes for 24 h (DON) **(B)** or 9 h [15-acDON **(C)**, Fu-X **(D)**, or NIV **(E)**]. Results are shown as mean values ± SD and asterisks indicate significant differences from each vehicle group (*p < 0.05, **p < 0.01, ***p < 0.001, *n* = 3–4).

As a variety of signaling components for NF-κB activation are regulated by the ubiquitin-linked proteasome system, we assessed the effects of PSMA3 on p65 protein levels in response to 8-keto-trichothecenes in human IECs. PSMA3 deficiency did not significantly influence the total and phosphorylated p65 expression in the absence and presence of DON (Figure 4A). Similar patterns were also observed in response to other 8-keto-trichothecenes (15-acDON, Fu-X, and NIV; Figures 1A–C). Since treatment with 8-keto-trichothecens reduced total levels of p65 protein, the ratio of phospho-p65 to total p65 was not a representative index of NF-κB activation. Instead, we additionally calculated the ratio of phosphor-p65 to stable β-actin. Since DON reduced p65 mRNA levels (Figure 3C), we further measured the p65 transcription activity using cells expressing the human p65 promoter (−999/+92)-containing SEAP reporter. However, PSMA3 was not involved in the transcriptional regulation of p65 expression in response to DON (Figure 4B). Other 8-keto-trichothecenes including 15-acDON, Fu-X, and NIV also displayed suppressive effects on total and phosphorylated p65 protein levels in response to 8-keto-trichothecenes in a dose-dependent manner; however, these were not altered by PSMA3 deficiency in IECs (Figures 1A–C). Similarly, treatment with 15-acDON (Figure 4C), Fu-X (Figure 4D), or NIV (Figure 4E) suppressed the transcriptional activity for p65 expression, which was not affected by PSMA3 deficiency. Taken together, suppression of p65 expression by 8-keto-trichothecenes was independent of PSMA3 levels whereas PSMA3 counteracted the EGR-1 levels in response to 8-keto-trichothecenes in human IECs.

### Dual Reporter System Represents 8-keto-trichothecene-specific Patterns of Inflammatory Regulation

**Figure 5:**
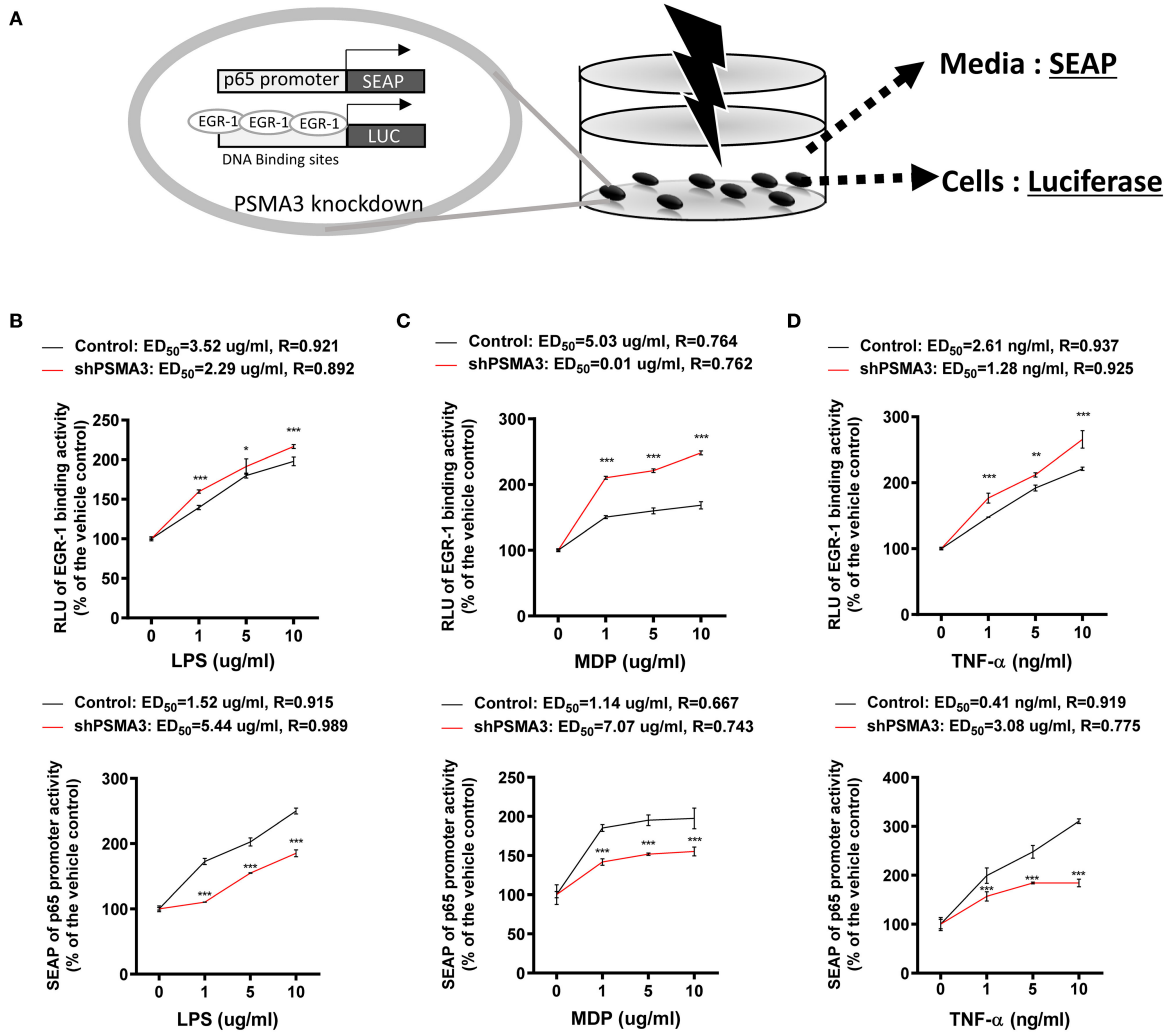
Effects of inflammagens on the dual reporter system in human IECs. **(A)** A putative scheme for the mechanism of the dual reporter system (SEAP for p65 transcription and luciferase for EGR-1-mediated transcription) to detect 8-keto-trichothecenes in PSMA3-deficient IECs. **(B)** Control- or PSMA3-deficient HCT-8 with dual reporters were treated with each dose of LPS **(B)**, MDP **(C)**, or TNF-α **(D)** for 72 h. Levels of SEAP in media and intracellular luciferase were simultaneously measured after exposure. Results are shown as mean values ± SD and asterisks indicate significant differences from the control group at each dose (*p < 0.05, **p < 0.01, ***p < 0.001, *n* = 3).

**Figure 6:**
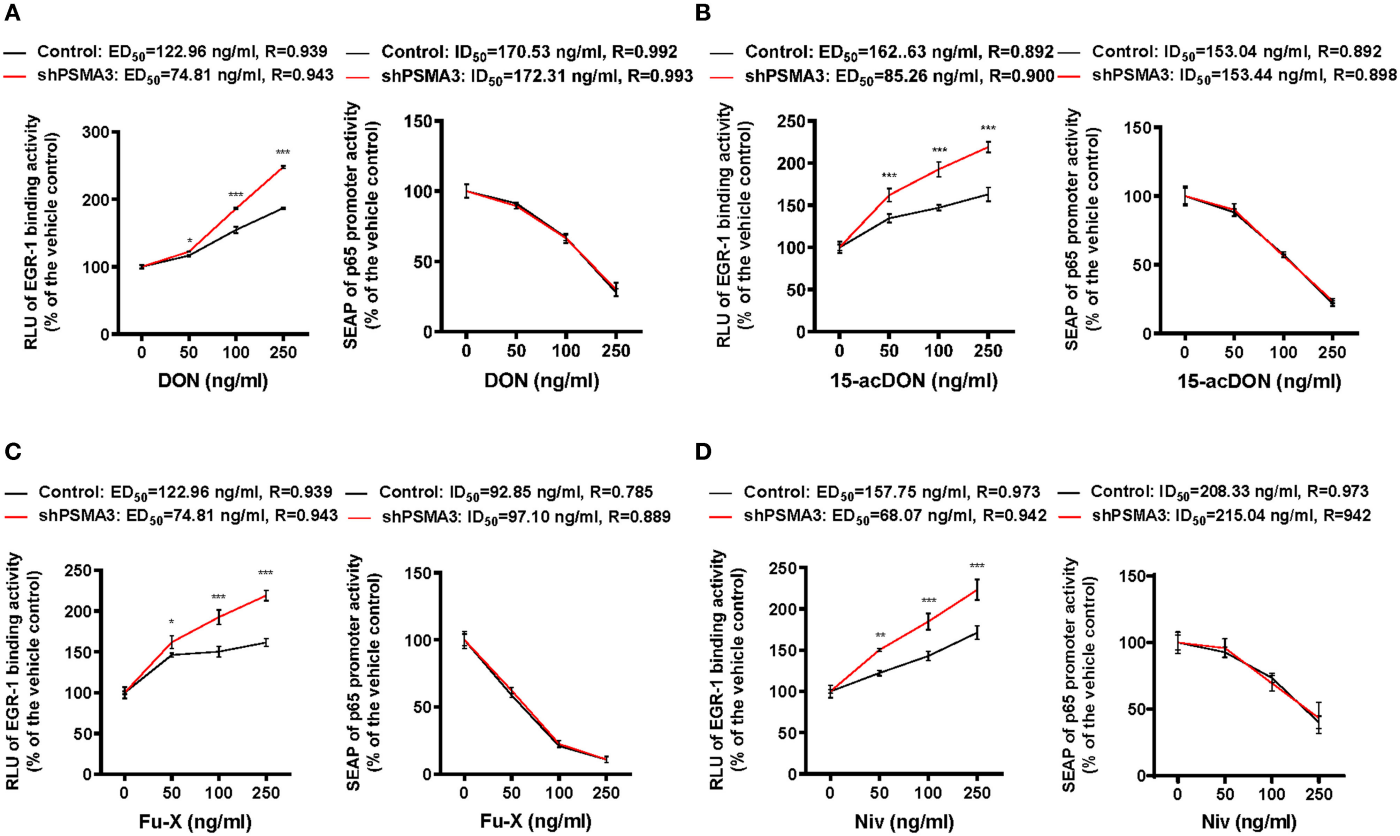
Effects of 8-keto-trichothecenes on the dual reporter system in human IECs. Control- or PSMA3-deficient HCT-8 with dual reporters were treated with each dose of DON **(A)**, 15-acDON **(B)**, Fu-X **(C)**, or NIV **(D)** for 24 h. Levels of SEAP in media and intracellular luciferase were simultaneously measured after exposure. Results are shown as mean values ± SD and asterisks indicate significant differences from the control group at each dose (*p < 0.05, **p < 0.01, ***p < 0.001, *n* = 3).

We determined that 8-keto-trichothecenes induced EGR-1 while suppressing p65 promoter activity in IECs. In addition, PSMA3 deficiency enhanced the EGR-1 levels in response to 8-keto-trichothecenes in IECs. Based on these mechanistic evidences, we established a cell-based bioassay system representing the proinflammatory actions of 8-keto-trichothecenes in IECs. Control and PSMA3-deficient cells were stably transfected with dual reporter plasmids including pEBS1^4^-LUC and a human p65 promoter (−999/+92)-containing SEAP reporter plasmid. The pEBS1^4^-LUC plasmid was constructed to detect EGR-1-mediated transcriptional activity whereas SEAP in medium was assessed to quantify p65 promoter activity in response to inflammagens (Figure 5A). We also performed the dual reporter bioassay in response to a cytokine or bacterial endotoxin as positive proinflammatory triggers. We calculated the effective doses for enhancing reporter production by 50% (ED_50_) or the inhibitory doses for reducing reporter production by 50% (ID_50_) in the dual reporter system. In response to bacterial cell wall components including LPS or MDP (the minimal bioactive peptidoglycan motif), or tumor necrosis factor-α (TNF-α), both EGR-1-mediated transcription and p65 promoter activities were elevated in a dose-dependent manner (Figures 5B–D). Moreover, PSMA3 deficiency improved the dose responses of EGR-1-mediated transcription to the conventional proinflammatory triggers, particularly MDP, compared to those in the controls. In contrast, PSMA3 deficiency attenuated the p65 promoter activation in response to three inflammagens, indicating that PSMA3 is a positive regulator of NF-κB signaling activation by endotoxins or TNF-α. In response to 8-keto-trichothecenes, EGR-1-mediated transcription was elevated whereas p65 promoter activity was suppressed in a dose-dependent manner (Figure 6). Furthermore, the sensitivity of EGR-1-mediated transcription in response to 8-keto-trichothecenes was enhanced by PSMA3 deficiency. However, the half-inhibitory dose (ID_50_) for p65 promoter activity was not significantly altered by PSMA3 deficiency in the presence of 8-keto-trichothecenes. In conclusion, the dual reporter system in PSMA3-deficient IECs was promising for the biomonitoring of typical inflammatory regulation by 8-keto-trichothecenes, which was differentiated by the responses to conventional inflammatory triggers including endotoxins and cytokines.

## Discussion

Based on mechanistic evidences on the regulation of proinflammatory transcription factors by 8-keto-trichothecenes, we developed an IEC-based biomonitoring method to detect the biological activity of trichothecene mycotoxins. Trichothecene-induced chemokine production is regulated by EGR-1 or NF-κB in IECs, which was simulated using cells with dual reporters for these two transcription factors. Moreover, the sensitivity of EGR-1-based biomonitoring was improved by suppressing PSMA3, the α-type subunit of the 20S proteasome core complex. However, PSMA3 was positively involved in p65 promoter response to endotoxins and cytokines. Mechanistically, NF-κB-linked signaling pathways are strongly associated with ubiquitin-proteasome regulation. In particular, ubiquitination reactions of TNF receptor associated factor (TRAF6), IκB kinase γ (IKKγ/NEMO), and Iκα contribute to NF-κB activation via regulation of protein binding or degradation during signaling transduction (50, 51). Despite no known evidences in NF-κB regulation by PSMA3, the present study first identified PSMA3 as a positive regulator of p65-linked signaling in IECs. PSMA3 deficiency decreased the responses of p65-linked promoter activity to conventional inflammagens including endotoxins and TNFα. However, PSMA3 did not contribute to the regulation of p65 expression in response to 8-keto-trichothecenes.

Although treatment with 8-keto-trichothecenes enhanced production of proinflammatory chemokines, it downregulated the key transcription factor NF-κB in gut epithelial cells. Instead, EGR-1 was mainly involved in transcription activation of proinflammatory chemokine genes in the present exposure model. Moreover, our previous investigation demonstrated that DON-induced EGR-1 mediates the induction of peroxisome proliferator-activated receptor γ (PPARγ), which is associated with suppression of pro-inflammatory NF-κB signaling (39). Mechanistically, PPARγ attenuates inflammatory responses by triggering nuclear export of p65 protein in complex with PPARγ (52). Moreover, as another transcriptional regulator of proinflammatory signaling, activating transcription factor 3 (ATF3) is induced as part of the negative feedback loop that modulates pattern recognition receptor-stimulated inflammatory responses (53–55). ATF3 is also induced by DON as part of a transcriptional complex composed of the NF-κB family of transcription factors (29). ATF3 can alter the chromatin structure, thereby restricting access to other transcription factors including NF-κB (53). However, treatment with 8-keto-trichothecenes reduced total levels of p65 protein in human intestinal epithelial cells, which may occur in advance of promoter-binding regulation of NF-κB by either ATF3 or PPARγ. One potent explanation could be a post-transcriptional regulation of p65 since DON treatment downregulates the stability of p65 mRNA (49). In particular, ATF3 leads to the destabilization of p65 mRNA caused by nuclear entrapment of transcript-stabilizing HuR protein via direct interaction with ATF3 (49). In addition to ATF3, PPARγ is a negative regulator of transcript stability since it suppresses the cytoplasmic translocation of HuR (56), which may contribute to reduction in p65 protein in response to 8-keto-trichothecenes in the present study. Suppression of NF-κB could be a beneficial event during inflammation, but which can interfere with the tissue regeneration after injuries since epithelial NF-κB is involved in cellular proliferation and migration (57–60). Thus, it can be speculated that the wound healing process during toxin-induced inflammation can be interfered due to NF-κB suppression by DON. While trichothecene-induced EGR-1 plays pivotal roles in proinflammatory cytokine production, attenuated NF-κB signaling may account for delayed epithelial restitution after gut injury.

The present bioassay system provides high sensitivity to detect 8-keto-trichothecenes, which can be detected at very low doses (<0.1 ppm), far less than the general regulatory limit of 1 ppm. Moreover, the present bioassay system represents the quantitative biological actions of trichothecenes. Until now, most of eukaryotic cell-based bioassays of trichothecenes are based on the detection of the inhibitory effects of the mycotoxins on protein synthesis, secretion, or cell proliferation (13, 61–63). However, the present bioassay indicates the typical biological processes regulated by inflammation-associated transcription factors including EGR-1 and NF-κB in human intestinal epithelial cells. Trichothecene-exposed cells displayed opposite readout patterns of EGR-1 and p65, both of which were elevated by endotoxins and TNF-α. In addition to these two factorials (EGR-1 and p65), regulation of PSMA3 contributed to the enhanced specificity of the dual reporter system. These combinatorial features of responses can indicate the biological identity of 8-keto-trichothecenes in the tested matrix in the present biomonitoring system. Moreover, most (99%) cereal foodstuffs are contaminated with more than 10 mycotoxins. Mixtures of 8-keto-trichothecenes are often found in field crops depending on weather conditions and mold chemotypes. Further, 10–20% of DON and NIV exist in the acetylated form such as 3-acetyl DON, 15-acetyl DON, or Fu-X. For efficient risk evaluation of the 8-keto-trichothecene mixture, the total quantity of the biological actions of toxin mixtures should be calculated based on the mechanism along with the chemical identification. Therefore, the relative risk of chemicals with a common mechanism of action can be assessed by measuring readouts such as the toxic equivalent factor (TEF) through the standard bioassay (13, 64). For example, based on toxin-induced actions such as endoplasmic reticulum stress, a reporter-based TEF can be derived by comparing the ED_50_ or ID_50_ of each 8-keto-trichothecene (13). Moreover, TEF-based quantitative and qualitative assessment contributes to the risk characterization such as the total daily intake by humans and animals. The present bioassay using typical patterns of proinflammatory signaling can be standardized with TEF evaluation and further integration for regulatory and industrial uses.

The present bioassay system could be a potential quantitative application for the immunotoxicological determination of 8-keto-trichothecene mixtures in agricultural commodities. However, it needs to be improved for *in vitro* recapitulation of the realistic pathophysiological gut environment. For instance, the intoxicated tissue contexture is different from two-dimensional culture of intestinal epithelial cells. Local adverse outcomes can be differentiated depending on the accessibility of toxins to epithelial surface in the gut. Therefore, *in vivo* gut-mimicking culture of three-dimensional monolayer such as intestinal organoids would provide more realistic information on trichothecene stress responses than the simple monolayer model (65, 66). Moreover, dose responses in the static culture model hardly represent the toxicokinetic responses in animals and humans. In particular, local luminal concentration of trichothecenes in the gastrointestinal tract following ingestion is generally much higher than levels in the circulation and other deposition tissues. Moreover, while the proximal parts of the small intestine are the major site of DON absorption (67), DON rarely reaches the distal small intestine and colon at the luminal region. Instead, the distal parts are mainly affected by circulating DON via the basolateral exposure (68). Therefore, additional epithelial readouts from the mucosal signature (69) are warranted to simulate compartment-specific actions of 8-keto-trichothecenesin the gut.

## Data Availability Statement

The raw data supporting the conclusions of this article will be made available by the authors, without undue reservation, to any qualified researcher on reasonable request.

## Ethics Statement

The animal study was reviewed and approved by Pusan National University Institutional Animal Care and Use Committee (PNU-IACUC) (PNU-2013-0291).

## Author Contributions

Project design and hypotheses were defined by YM and S-HP. S-HP conducted experiments and analyzed the data. YM and S-HP prepared the manuscript. YM supervised the overall project. All authors contributed to the article and approved the submitted version.

## Conflict of Interest

The authors declare that the research was conducted in the absence of any commercial or financial relationships that could be construed as a potential conflict of interest.
